# Single-cell RNA sequencing reveals cell subpopulations in the tumor microenvironment contributing to hepatocellular carcinoma

**DOI:** 10.3389/fcell.2023.1194199

**Published:** 2023-06-02

**Authors:** Jiamin Gao, Zhijian Li, Qinchen Lu, Jialing Zhong, Lixin Pan, Chao Feng, Shaomei Tang, Xi Wang, Yuting Tao, Jianyan Lin, Qiuyan Wang

**Affiliations:** ^1^ Department of Biochemistry and Molecular Biology, School of Basic Medical Sciences, Guangxi Medical University, Nanning, China; ^2^ Center for Genomic and Personalized Medicine, Guangxi Medical University, Nanning, China; ^3^ Laboratory of Infectious Disease, The Fourth People’s Hospital of Nanning, Nanning, China; ^4^ Department of Clinical Laboratory, The First Affiliated Hospital of Guangxi Medical University, Nanning, China; ^5^ Guangxi Key Laboratory for Genomic and Personalized Medicine, Guangxi Collaborative Innovation Center for Genomic and Personalized Medicine, Nanning, China; ^6^ Administrative Office, The First People’s Hospital of Nanning, Nanning, China

**Keywords:** hepatocellular carcinoma, single-cell RNA sequencing, tumor microenvironment, receptor-ligand interaction, cellular communication

## Abstract

**Background:** Hepatocellular carcinoma (HCC) is among the deadliest cancers worldwide, and advanced HCC is difficult to treat. Identifying specific cell subpopulations in the tumor microenvironment and exploring interactions between the cells and their environment are crucial for understanding the development, prognosis, and treatment of tumors.

**Methods:** In this study, we constructed a tumor ecological landscape of 14 patients with HCC from 43 tumor tissue samples and 14 adjacent control samples. We used bioinformatics analysis to reveal cell subpopulations with potentially specific functions in the tumor microenvironment and to explore the interactions between tumor cells and the tumor microenvironment.

**Results:** Immune cell infiltration was evident in the tumor tissues, and *BTG1* ^+^ *RGS1* ^+^ central memory T cells (Tcms) interact with tumor cells through *CCL5*-*SDC4/1* axis. HSPA1B may be associated with remodeling of the tumor ecological niche in HCC. Cancer-associated fibroblasts (CAFs) and macrophages (TAMs) were closely associated with tumor cells. *APOC1* ^+^ *SPP1* ^+^ TAM secretes SPP1, which binds to ITGF1 secreted by CAFs to remodel the tumor microenvironment. More interestingly, *FAP* ^+^ CAF interacts with naïve T cells via the *CXCL12–CXCR4* axis, which may lead to resistance to immune checkpoint inhibitor therapy.

**Conclusion:** Our study suggests the presence of tumor cells with drug-resistant potential in the HCC microenvironment. Among non-tumor cells, high *NDUFA4L2* expression in fibroblasts may promote tumor progression, while high *HSPA1B* expression in central memory T cells may exert anti-tumor effects. In addition, the *CCL5–SDC4/1* interaction between *BTG1* ^+^ *RGS1* ^+^ Tcms and tumor cells may promote tumor progression. Focusing on the roles of CAFs and TAMs, which are closely related to tumor cells, in tumors would be beneficial to the progress of systemic therapy research.

## 1 Introduction

Liver cancer is the third most common cause of cancer-related death worldwide, and hepatocellular carcinoma (HCC) accounts for 90% of primary liver cancer (Cunha et al., 2021). HCC is one of the deadliest cancers worldwide and has become an increasingly major and growing health problem in both developing and developed countries. It has a variety of causes, including liver cirrhosis, chronic infection with hepatitis B or C virus, excessive alcohol consumption, type 2 diabetes mellitus, non-alcoholic fatty liver disease, non-alcoholic steatohepatitis, exposure to the environmental toxicant aflatoxin-B1, obesity, and metabolic syndrome (D'Souza et al., 2020; Kumar et al., 2021; Thylur et al., 2020). Currently, hepatic resection and liver transplantation are the main treatments for early-stage HCC; however, most diagnoses are already in the progressive stage, where the opportunity for surgery has passed. Early recurrence within 2 years of resection accounts for 70% of recurrent HCC cases, and the difficulty of treatment after recurrence increases (Sun et al., 2021). Furthermore, liver transplantation faces the problem of insufficient liver supply. Thus, patients with advanced HCC often choose molecular targeted therapy and immune checkpoint inhibitor therapy; however, the latter is still in its infancy. More immune cell markers must be explored to predict the response of HCC to immune checkpoint inhibitor therapy.

Tumors are complex ecosystems involving malignant cells, immune cells, stromal cells, blood vessels, and the extracellular matrix (ECM) (Anderson and Simon, 2020). Tumor cells develop and evolve into a complex and tightly linked tumor microenvironment (TME) that affects tumor growth, metastatic spread, and response to therapy (Bilotta et al., 2022). Furthermore, liver fibrosis is a pathological process necessary for the progression of chronic liver disease into cirrhosis and HCC (Roehlen et al., 2020). The profibrotic program responds to tissue injury; however, persistent injury and damage can lead to dysregulation of this process, promoting myofibroblast activity and a chronic inflammatory environment with infiltrating macrophages and lymphocytes (Wynn, 2008). Tumor-associated macrophages (TAMs) are key components of the complex TME ecology and are influenced by tumor-derived cytokines, which exhibit significant immunosuppressive effects that promote the malignancy and progression of various tumors (Zhou et al., 2021). Cancer-associated fibroblasts (CAFs) are the most abundant stromal cells in the TME and are associated with cancer progression. An increasing number of studies have focused on interactions between CAFs and different immune cells in cancer. For example, CAFs regulate macrophages by secreting cytokines that promote cancer progression (An et al., 2020). However, the mechanism by which the TME in HCC contributes to its progression remains unclear. Understanding the interactions between cell types is essential for understanding tumor development, prognosis, and treatment.

Single-cell RNA sequencing (scRNA-seq) is a powerful tool for studying cellular components and their interactions within the TME. In this study, a single-cell profile of HCC was constructed using scRNA-seq, revealing the differences in cell-type composition in the HCC TME and elucidating the interactions between tumor cells and the TME.

## 2 Materials and methods

### 2.1 Data sources

The HCC scRNA-seq data were obtained from Gene Expression Omnibus (GEO) database GSE156625 (Platform: GPL16791) (Sharma et al., 2020). A total of 43 tumor tissue samples and 14 adjacent control samples from 14 patients with HCC were included in this study. Healthy normal liver samples, fetal liver samples, and mouse liver samples were excluded. In addition, Cancer Genome Atlas Liver Hepatocellular Carcinoma (TCGA-LIHC) data were obtained from the TCGA database (https://portal.gdc.cancer.gov/projects/TCGA-LIHC), including 374 HCC tissues with bulk RNA-seq data and the corresponding clinical information.

### 2.2 Construction of the single-cell atlas

Single-cell data were processed in quality control, filtering the 1% of cells with the highest and lowest feature numbers of expression, as well as cells with more than 10% mitochondrial gene expression. After quality control, the data were integrated and analyzed based on SCTransform standardization (Hafemeister and Satija, 2019). The merged data were subjected to cell clustering analysis using the default parameters of the R package Seurat (Butler et al., 2018). A single-cell atlas was constructed as described previously (Liang et al., 2022b). Cell clusters were first identified based on the FindNeighbors and FindClusters functions of the Seurat package, and then downscaled and visualized as a single-cell atlas based on the Unified Modal Approximation and Projection (UMAP) algorithm (Becht et al., 2018). Cluster-specific marker genes were identified using the FindAllMarkers function of the Seurat package, and cell types were identified based on the expression of highly specific genes in the cell clusters as well as classical cell markers.

### 2.3 Copy number variation analysis

The copy number variations (CNVs) in tumor cells derived from the HCC tissues were calculated using the R package inferCNV (v1.6.0; inferCNV of the Trinity CTAT Project, https://github.com/broadinstitute/inferCNV) to identify subclones of diseased cells and to infer tumor evolution.

### 2.4 Functional enrichment analysis

To explore biological status and functional differences between different cell subpopulations, we performed functional enrichment analysis based on the Gene Ontology (GO) and Kyoto Encyclopedia of Genes and Genomes (KEGG) databases using the R package clusterProfiler (Yu et al., 2012). The hypergeometric test was used to compare differences, and *p* < 0.05 was considered statistically significant.

### 2.5 Construction of gene regulatory networks

Gene regulatory networks (GRNs) determine and maintain cell-type-specific transcriptional states; active transcription factors (TFs) and their target genes are commonly denoted as GRNs. To explore the key regulators that drive and maintain cellular state behavior, we constructed GRNs with TFs as their cores, divided them into several groups of co-expression modules, and inferred GRNs and cell states based on single-cell expression profiles with single-cell regulatory network inference and clustering (SCENIC) (Van de Sande et al., 2020). The binding patterns of the TFs were obtained from the JASPAR database (https://jaspar.genereg.net).

### 2.6 Pseudotime analysis

Development or external stimuli can change the functions of different cells in different ways, resulting in differential gene expression within the cells. Sorting these cells according to gene expression enabled further inference of cell development trajectory. In this study, the cell lineage trajectory of each cell type was inferred using Monocle3 (Trapnell et al., 2014), and the cells were projected into a low-dimensional space using UMAP.

### 2.7 Cell communication analysis

Tumor cells can interact with surrounding cells through the circulatory and lymphatic systems, thereby influencing the development and progression of cancer. Thus, to further investigate cellular interactions in the HCC microenvironment, we used the R package iTALK for cellular communication analysis. iTALK identifies high-confidence ligand–receptor interactions between cells by identifying genes that are highly or differentially expressed in cell clusters and matching these genes to a built-in ligand–receptor database (Wang et al., 2019).

### 2.8 Receiver operating characteristic curve and survival analysis

The R package limma (Ritchie et al., 2015) was used to normalize the TCGA-LIHC data. Receiver operating characteristic (ROC) curve analysis was performed using the R package pROC (Robin et al., 2011). In addition, to further explore the prognostic potential of highly expressed genes specific to each cell subpopulation, overall survival (OS) and recurrence-free survival (RFS) analyses of HCC were performed using the R package Survminer (https://rdocumentation.org/packages/survminer), and differences were calculated using the log-rank test with *p* < 0.05 considered significant.

## 3 Results

### 3.1 The global landscape of the HCC immune microenvironment

**FIGURE 1:**
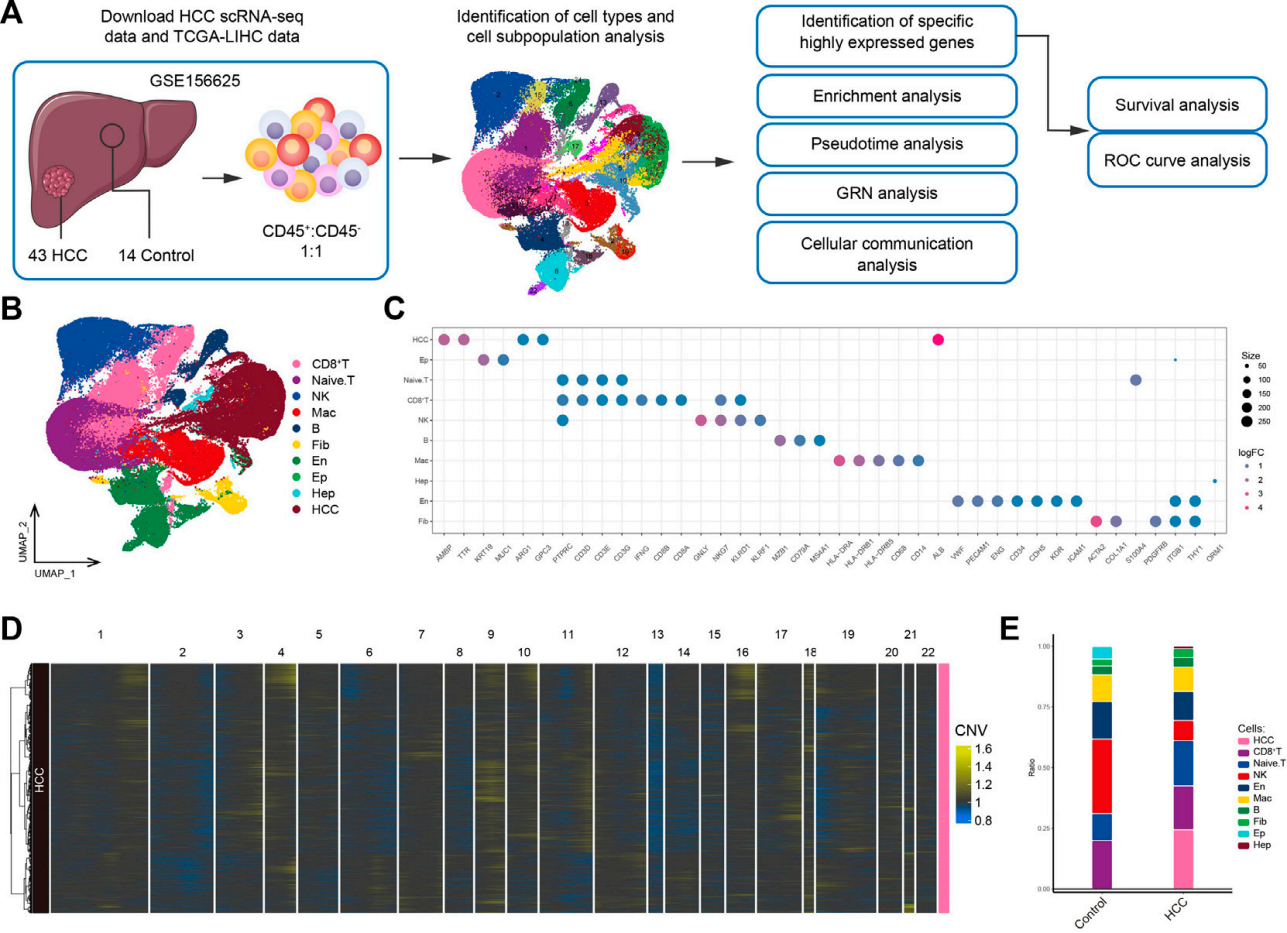
Single-cell transcriptome profiling of patients with HCC. **(A)** Single-cell analysis process. **(B)** Global HCC tumor ecological map showing the different cell types. **(C)** Expression of marker genes for cellular annotation (size: -log10 (adjusted *p*-value); statistical test method: Wilcoxon rank sum). **(D)** The chromosomal landscape of HCC tumors. **(E)** The difference in composition of 10 cell types between the control and HCC groups. HCC: Hepatocellular carcinoma; TCGA-LIHC: the Cancer Genome Atlas Liver Hepatocellular Carcinoma data collection; GRN: gene regulatory network; ROC: receiver operating characteristic.

The workflow of the study is shown in Figure 1A. To investigate the tumor ecological landscape in patients with primary HCC, we analyzed scRNA-seq data from GSE156625, which consisted of 43 tumor tissue samples and 14 adjacent control samples from 14 patients with HCC. After cell clustering and downscaling, 112,317 cells were grouped into 25 clusters manually identified according to 10 cell types with expressed markers consistent with previous laboratory and scRNA-seq studies (Sharma et al., 2020; Sun et al., 2021; Liang et al., 2022a) (Figure 1B), including HCC cells (*AMBP*, *TTR*, *ARG1*, *GPC3*, and *ALB*), hepatocyte (Hep; *ORM1*), epithelial cells (Ep; *KRT19* and *MUC1*), endothelial cells (En; *VWF*, *PECAM1*, *ENG*, *CD34*, *CDH5*, *KDR*, and *ICAM1*), fibroblasts (Fib; *ACTA2*, *COL1A1*, and *PDGFRB*), naïve T cells (*CD3D*, *CD3E*, and *CD3G*), CD8^+^ T cells (*IFNG*, *CD8A*, and *CD8B*), natural-killer cells (NK; *GNLY* and *KLRF1*), B cells (*MZB1*, *CD79A* and *MS4A1*), and macrophages (Mac; *HLA-DRA*, *HLA-DRB1*, *HLA-DRB5*, *CD68*, and *CD14*) (Figure 1C). The annotation of HCC cells was validated by inferring CNVs based on the scRNA-seq data (Figure 1D). Subsequently, by comparing the differences in cell type composition between HCC and control samples, we found naïve T cells to be more abundant in HCC than in adjacent control tissues (Figure 1E). This result suggested significant lymphatic immune infiltration of tumor cells. Additionally, the abundance of fibroblasts was higher and that of endothelial cells was lower in HCC than in adjacent control tissues. In summary, we constructed a single-cell atlas of patients with HCC, revealing differences in cell-type composition in the HCC tumor microenvironment.

### 3.2 Tumor cells with drug-resistant potential are present in the HCC microenvironment

**FIGURE 2:**
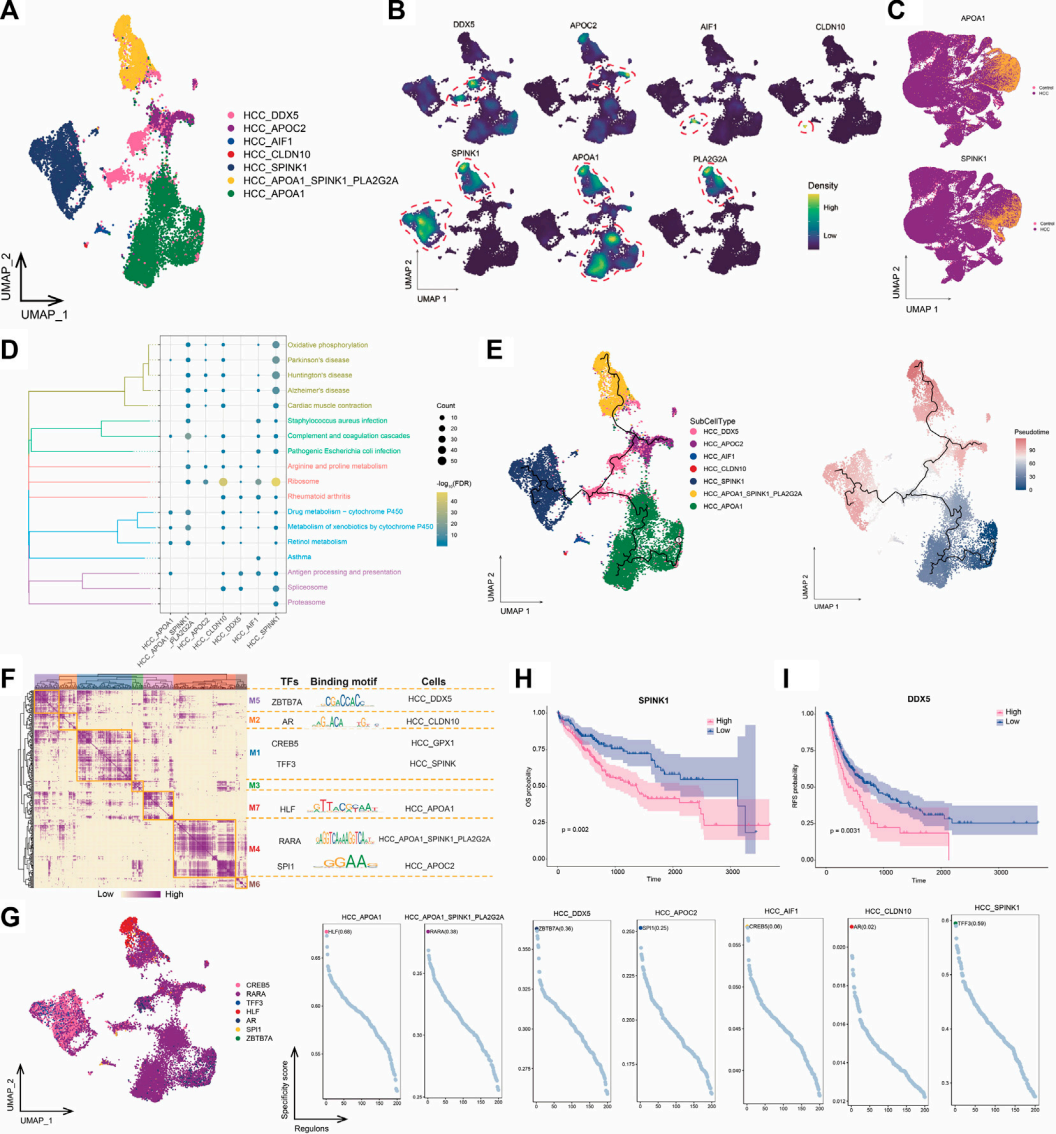
Landscape of tumor cell subpopulations in the HCC microenvironment. **(A)** HCC subpopulations identified in this study. **(B)** Markers that are specifically highly expressed in each HCC subpopulation. **(C)** Expression profiles of *APOA1* and *SPINK1* in the global single-cell landscape of HCC. **(D)** KEGG pathways significantly enriched in each HCC subpopulation. **(E)** Pseudotime differentiation trajectory of HCC subpopulations. **(F)** Gene regulatory network analysis of HCC subpopulations. **(G)** The main transcription factors regulating HCC subpopulations. **(H)** Kaplan-Meier plots showing worse OS prognosis in HCC with higher *SPINK1* expression. **(I)** Kaplan-Meier plots showing worse RFS prognosis in HCC with higher *DDX5* expression. HCC: hepatocellular carcinoma; OS: overall survival, RFS: relapse-free survival.

We performed a subpopulation analysis on the HCC cells and obtained seven subpopulations, including *APOA1* ^+^ HCC, *SPINK1* ^+^ HCC, *APOA1* ^+^ *SPINK1* ^+^ *PLA2G2A* ^+^ HCC, *DDX5* ^+^ HCC, *APOC2* ^+^ HCC, *AIF1* ^+^ HCC, and *CLDN10* ^+^ HCC (Figures 2A, B). Among these markers, *APOA1* was not only highly expressed in the *APOA1* ^+^ HCC subpopulation, but was also commonly expressed in other HCC subpopulations, and SPINK1 was also expressed in most HCC cells (Figure 2C). We conducted ROC curve analysis of *APOA1* and *SPINK1* based on the TCGA-LIHC data and found that high *APOA1* and *SPINK1* expression levels could serve as potential biomarkers for HCC, with AUC values of 0.782 and 0.690, respectively (Supplementary Figure S1). Enrichment analysis showed that *SPINK1* ^+^ HCC and *APOA1* ^+^ *SPINK1* ^+^ *PLA2G2A* ^+^ HCC cells were significantly involved in bacterial infection-related pathways (Figure 2D). Notably, all HCC subpopulations except *APOC2* ^+^ HCC were significantly involved in drug metabolism-related pathways, and most of these subpopulations were at the end of the cell developmental trajectory (Figure 2E). This result suggested that as HCC progresses, tumor cells may develop drug resistance by activating drug metabolism pathways, leading to poor outcomes in patients. Therefore, targeting these subpopulations could prevent the development of drug resistance in patients. We constructed GRNs to further explore the TFs regulating the subpopulations (Figures 2F, G). The results showed that subpopulations with drug-resistant potential were mainly regulated by TFs such as *CRZB5*, *AR*, *HLF*, and *RARA*, implying that tracking these genes is helpful for understanding HCC progression. In addition, we explored the prognostic potential of specific markers highly expressed in each subpopulation of HCC cells. The results suggested that high *SPINK1* and *DDX5* expression levels were significantly associated with lower HCC survival Figure 2H. In summary, drug metabolism pathways may be activated by tumor cells in the HCC microenvironment, resulting in increased difficulty in treatment.

### 3.3 High *NDUFA4L2* expression in fibroblasts may promote HCC development

**FIGURE 3:**
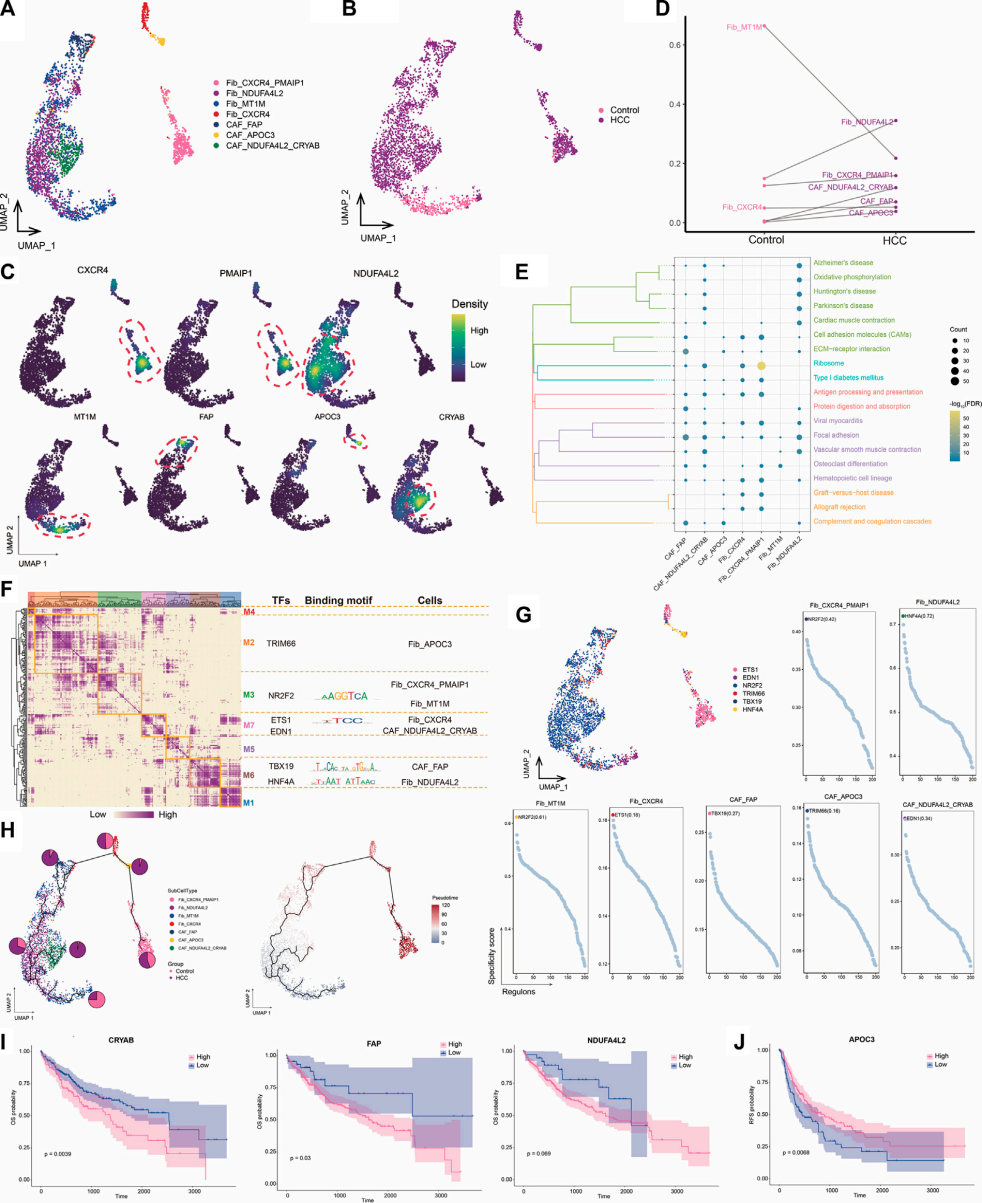
Landscape of fibroblast subpopulations in the HCC microenvironment. **(A)** Fibroblast subpopulations identified in this study. **(B)** Cellular profiles of fibroblast subpopulations in the control and HCC groups. **(C)** Markers that are specifically highly expressed in each fibroblast subpopulation. **(D)** Differences in the abundance of fibroblast subpopulations in the control and HCC groups. **(E)** KEGG pathways significantly enriched in each fibroblast subpopulation. **(F)** Gene regulatory network analysis of fibroblast subpopulations. **(G)** The main transcription factors regulating fibroblast subpopulations. **(H)** Pseudotime differentiation trajectory of fibroblast subpopulations. **(I)** Kaplan-Meier plots showing worse OS prognosis in HCC with higher expression of *CRYAB*, *FAP*, and *NDUFA4L2*. **(J)** Kaplan-Meier plots showing worse RFS prognosis in HCC with lower *APOC3* expression of. HCC: hepatocellular carcinoma; OS: overall survival, RFS: relapse-free survival.

CAFs are the most abundant cell type in the TME and are the center of cross-communication between various cells in the tumor mesenchyme. Thus, we performed a subpopulation analysis and identified seven major fibroblast subpopulations expressing different markers (Figures 3A–C). We found that the abundances of *NDUFA4L2* ^+^ Fib and *NDUFA4L2* ^+^ *CRYAB* ^+^ CAF cells were significantly increased in HCC, suggesting that these subpopulations may be involved in promoting tumor development (Figure 3D). Enrichment analysis revealed that all seven subpopulations were significantly associated with the focal adhesion pathway (Figure 3E), which is the point of contact between cells and the ECM and usually closely associated with cell migration. GRN analysis of the fibroblast subpopulations revealed that the target genes were divided into seven modules regulated by TFs including *NR2F2*, *ETS1*, *TBX19*, and *HNF4A* (Figures 3F, G). Ranking of the regulator specificity scores of the fibroblast subpopulations revealed that *NR2F2* was the most specific regulator of both the *CXCR4* ^+^ *PMAIP1* ^+^ Fib and *MT1M* ^+^ Fib subpopulations and has the ability to promote tumor cell proliferation, epithelial-mesenchymal transition, and invasive features (Mauri et al., 2021) (Figure 3G). As a gene therapy tool for HCC, forced re-expression of *HNF4A* inhibits proliferation and eliminates cancer-specific features of target cells (Takashima et al., 2018). Therefore, the combined transduction of *NR2F2* and *HNF4A* may be more stable for cellular reprogramming in HCC.

Pseudotime analysis showed that *NDUFA4L2* ^+^ Fib cells were positioned earlier on the differentiation trajectory than were *NDUFA4L2* ^+^ *CRYAB* ^+^CAF cells, presumably because *NDUFA4L2* ^+^ Fib cells differentiated into *NDUFA4L2* ^+^ *CRYAB* ^+^CAFcells over time, further promoting tumor development (Figure 3H). Subsequently, we evaluated the prognostic potential of specific markers highly expressed in each subpopulation of HCC and found that patients with higher *CYRAB*, *FAP*, and *NDUFA4L2* expression had poorer OS (Figure 3I), whereas patients with lower *APOC3* expression had poorer RFS (Figure 3J). Therefore, high *NDUFA4L2* expression in fibroblasts may promote HCC development. To address the location of fibroblasts in the TME, we need to further investigate their connections with other cells.

### 3.4 High *SLC9A3R2* expression in endothelial cells differentiates into other stages of En cells as HCC develops

**FIGURE 4:**
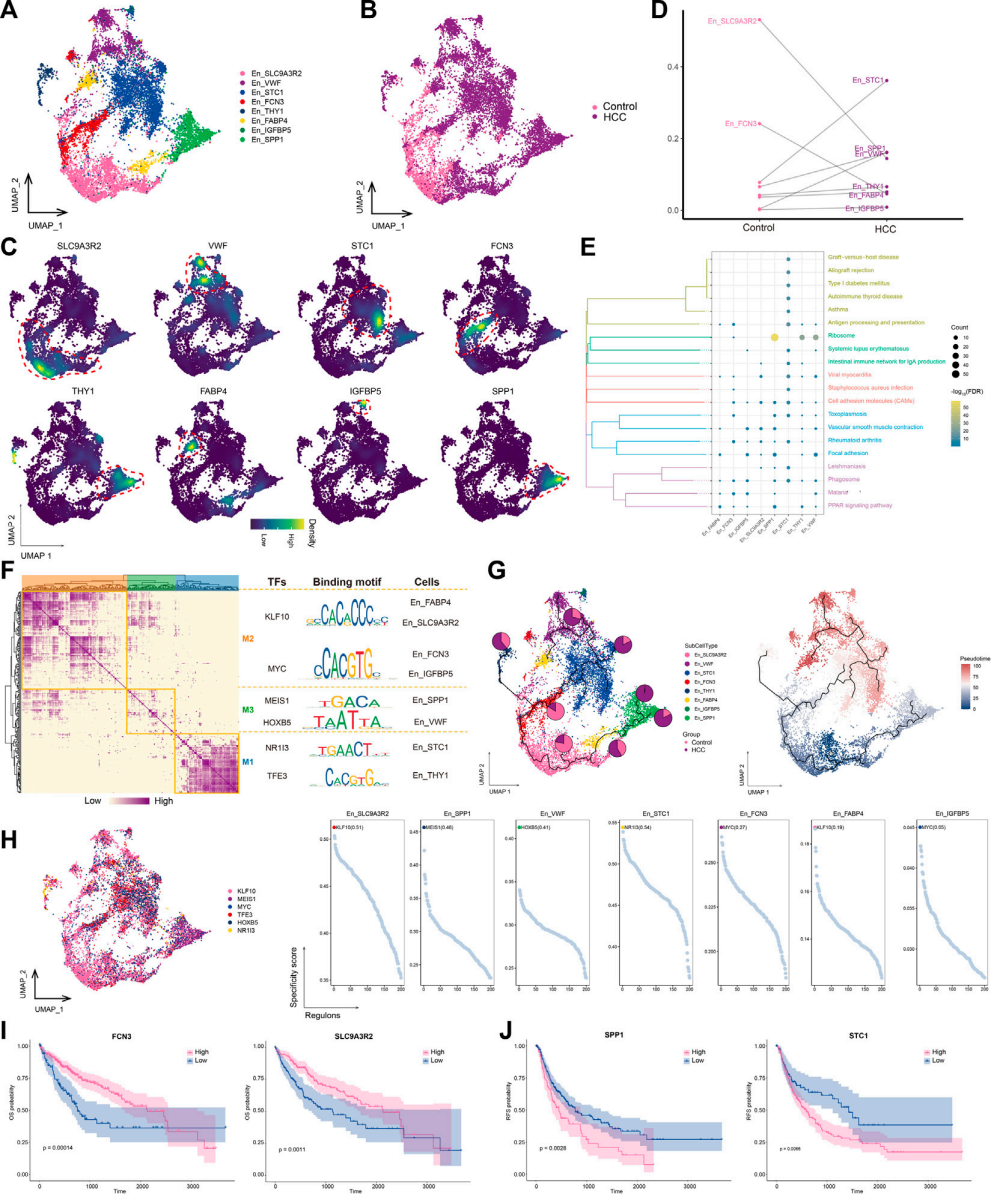
Landscape of endothelial cell subpopulations in the HCC microenvironment. **(A)** Endothelial cell (En) subpopulations identified in this study. **(B)** Cellular profiles of En subpopulations in the control and HCC groups. **(C)** Markers that are specifically highly expressed in each En subpopulation. **(D)** Differences in the abundance of En subpopulations in the control and HCC groups. **(E)** KEGG pathways significantly enriched in each En subpopulation. **(F)** Gene regulatory network analysis of En subpopulations. **(G)** The main transcription factors regulating En subpopulations. **(H)** The pseudotime differentiation trajectory of En subpopulations. **(I)** Kaplan-Meier plots showing worse OS prognosis in HCC with lower expression of *SLC9A3R2* and *FCN3*. **(J)** Kaplan-Meier plots showing worse RFS prognosis in HCC with lower expression of *SLC9A3R2* and *FCN3*. HCC: hepatocellular carcinoma; OS: overall survival, RFS: relapse-free survival.

The transformation of endothelial cells into mesenchymal fibroblasts after stimulation, known as the endothelial-mesenchymal transition (EMT), is an important source of fibroblasts. Our analysis confirmed the increased abundance of fibroblasts and decreased abundance of endothelial cells in the HCC group (Figure 1E). Thus, we performed a subpopulation analysis of the endothelial cells and obtained eight subpopulations (Figures 4A, B), named according to the markers they specifically expressed (Figure 4C). The abundances of *STC1* ^+^ En and *VWF* ^+^ Encells were significantly increased in the HCC group (Figure 4D), and *STC1* ^+^ En was the most abundant subtype. Most endothelial cell subpopulations were significantly associated with focal adhesion and cell adhesion molecule pathways (Figure 4E). GRN analysis of the endothelial cells revealed that the target genes were divided into three modules and regulated by TFs including *KLF10*, *MEIS1*, *HOXB5*, *NR1I3*, and *TFE3* (Figure 4F). Among them, KLF10 was the most specific regulator of two subpopulations, *FABP4* ^+^ En and *SLC9A3R2* ^+^ En, and has important regulatory effects on perivascular fibrosis (Zhuang et al., 2022) (Figure 4G). Moreover, the *SLC9A3R2* ^+^ En subpopulation, whose abundance was significantly reduced in HCC (Figure 4C), was not only significantly enriched in cell adhesion molecule pathways, but was also at an early stage of subpopulation cell differentiation, suggesting that it may gradually differentiate into other endothelial cell stages with the progression of HCC, in turn promoting tumor proliferation and migration (Figure 4H). Therefore, targeting *KLF10* to regulate the abundance of the *SLC9A3R2* ^+^ En subpopulation may help regulate HCC proliferation and migration. Survival analysis of marker genes showed that patients with low *SLC9A3R2* and *FCN3* expression had poorer OS (Figure 4I) and RFS (Figure 4J). Overall, these results suggest that *SLC9A3R2* ^+^ En cells differentiate into other endothelial cell stages as HCC progresses and contribute to its further development.

### 3.5 Increased infiltration of HCC tissues by macrophages highly expressing *APOC1*

**FIGURE 5:**
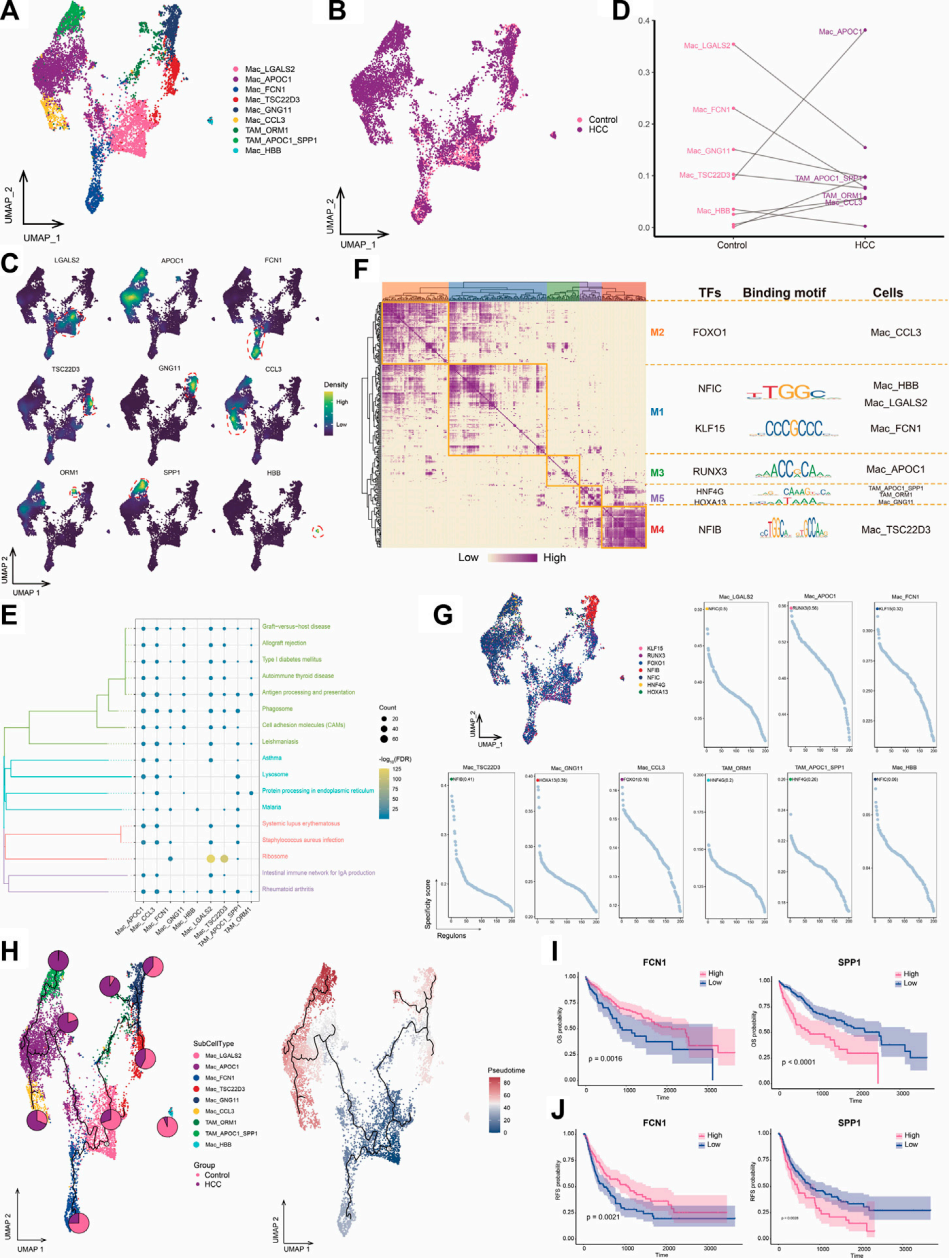
Landscape of macrophage subpopulations in the HCC microenvironment. **(A)** Macrophage subpopulations identified in this study. **(B)** Cellular profiles of macrophage subpopulations in the control and HCC groups. **(C)** Markers that are specifically highly expressed in each macrophage subpopulation. **(D)** Differences in the abundance of macrophage subpopulations in the control and HCC groups. **(E)** KEGG pathways significantly enriched in each macrophage subpopulation. **(F)** Gene regulatory network analysis of macrophage subpopulations. **(G)** The main transcription factors regulating macrophage subpopulations. **(H)** Pseudotime differentiation trajectory of macrophage subpopulations. **(I)** Kaplan-Meier plots showing the relationship between OS prognosis in *HCC* and *FCN1* and *SPP1* expression. **(J)** Kaplan-Meier plots showing the relationship between RFS prognosis in *HCC* and *FCN1* and *SPP1* expression. HCC: hepatocellular carcinoma; OS: overall survival, RFS: relapse-free survival.

TAMs are macrophages that infiltrate tumor tissues and exert tumor-promoting and immunosuppressive effects in various ways (Wium et al., 2018; Gadiyar et al., 2020; Hedrich et al., 2021). We identified nine macrophage subpopulations and subsequently assigned names based on genes specifically highly expressed in these subpopulations (Figures 5A–C). Among them, the *APOC1* ^+^ Mac subpopulation was a major component of HCC macrophage ecology compared to levels in adjacent control tissue, whereas the abundance of the *FCN1* ^+^ Mac subpopulation was significantly decreased in tumor cells. The *APOC1* ^+^ *SPP1* ^+^ TAM and *HSPA1B* ^+^ TAM subpopulations were particularly abundant in the HCC group and were therefore defined as TAMs (Figure 5D). Most macrophage subpopulations were significantly involved in pathways related to antigen processing and expression, suggesting an active immune function of macrophages in the HCC microenvironment (Figure 5E). GRN analysis of macrophages revealed that the target genes were divided into five modules regulated by TFs including *NFIC*, *KLF15*, *RUNX3*, *HNF4G*, *HOXA13*, and *NFIB* (Figures 5F, G). Pseudotime analysis showed similar differentiation trajectories for *APOC1* ^+^ Mac and *APOC1* ^+^ *SPP1* ^+^ TAM cells, which were significantly more abundant in HCC, suggesting increased infiltration by macrophages highly expressing *APOC1* with the development of HCC (Figure 5H). Macrophage subpopulations with high *APOC1* expression were found at the end of the cell developmental trajectory and specifically expressed the M2 macrophage marker CD163 (Supplementary Figure S2), suggesting that these subpopulations may accelerate HCC development. Survival analysis of the subpopulation-specific markers showed that *FCN1* and *SPP1* had prognostic potential for HCC (Figures 5I, J). In HCC, the presence of TAM subpopulations correlates with poor outcomes (Graham and Pollard, 2022). In our study, TAM subpopulations were positioned at the end of the cell developmental trajectory and were regulated by the same TFs, implying that macrophage cell states could be changed to prevent the development of tumor cells.

### 3.6 Central memory T cells with high *HSPA1B* expression play a significant role in HCC immunity

**FIGURE 6:**
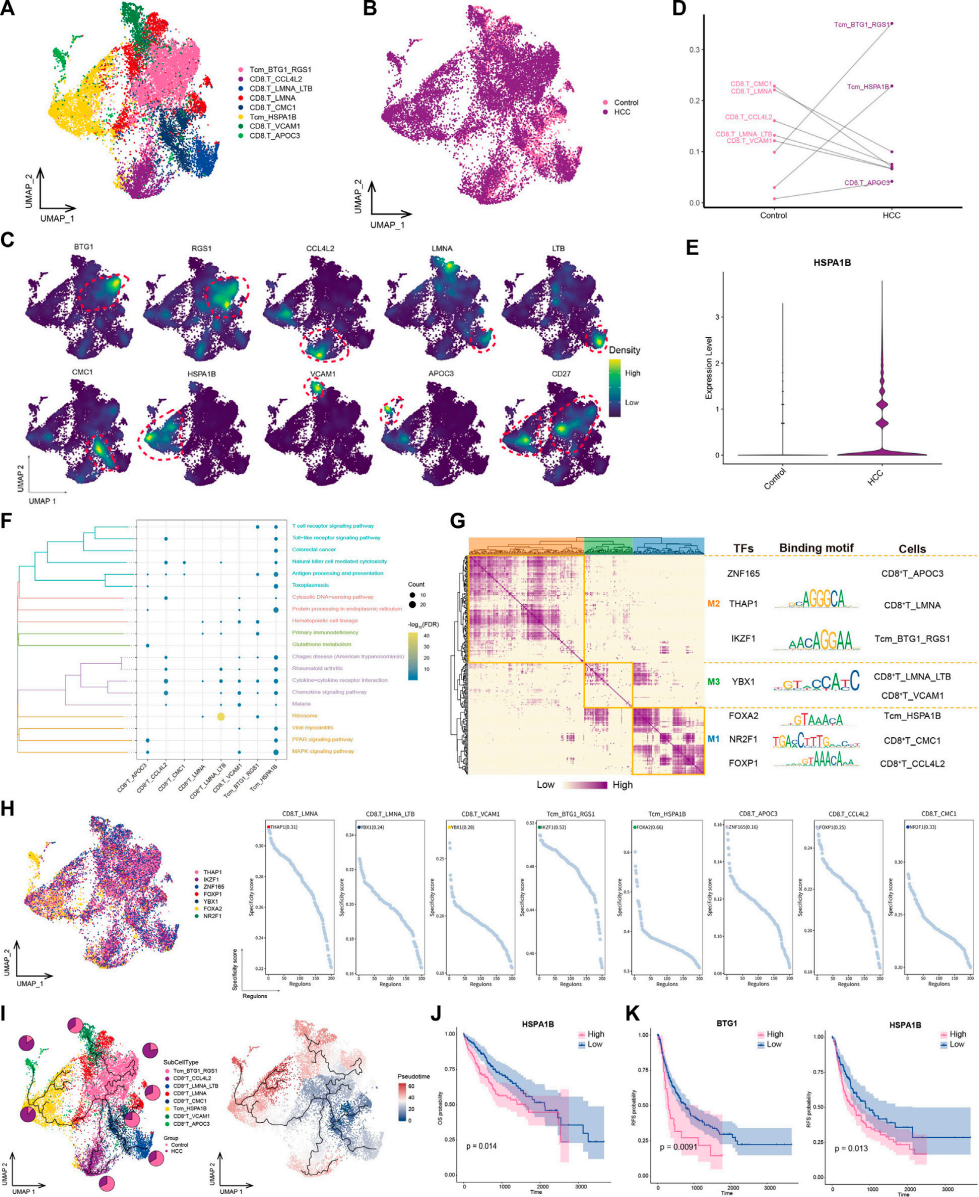
Landscape of CD8^+^ T subpopulations in the HCC microenvironment. **(A)** CD8^+^ T subpopulations identified in this study. **(B)** Cellular profiles of CD8^+^ T subpopulations in the control and HCC groups. **(C)** Markers that are specifically highly expressed in each CD8^+^ T subpopulation. **(D)** Differences in the abundance of CD8^+^ T subpopulations in the control and HCC groups. **(E)** Expression of HSPA1B in the control and HCC groups. **(F)** KEGG pathways significantly enriched in each CD8^+^ T subpopulation. **(G)** Gene regulatory network analysis of CD8^+^ T subpopulations. **(H)** The main transcription factors regulating CD8^+^ T subpopulations. **(I)** Pseudotime differentiation trajectory of CD8^+^ T subpopulations. **(J)** Kaplan-Meier plots showing worse OS prognosis in HCC with higher *HSPA1B* expression. **(K)** Kaplan-Meier plots showing worse RFS prognosis in HCC with higher *HSPA1B* and *BTG1* expression. HCC: hepatocellular carcinoma; OS: overall survival, RFS: relapse-free survival.

T lymphocytes play an important effector-killing role in antitumor immunity. To explore the differences between lymphocytes in tumors and normal tissues, we identified eight CD8^+^ T cell subpopulations (Figures 6A, B) expressing different specific markers (Figure 6C). Among them, CD27 was specifically expressed in the *BTG1* ^+^ *RGS1* ^+^ Tcm and *HSPA1B* ^+^ Tcm subpopulations, thus classifying them as subpopulations of central memory T cells (Tcms) (Figure 6E). The subpopulations with double-positive expression of *BTG1* and *RGS1* were significantly enriched in tumor lesions (Figure 6D). In addition, the *HSPA1B* ^+^ Tcm subpopulation was significantly enriched in the HCC group, and *HSPA1B* was significantly enriched in all cell types in the HCC group (Figure 6C), suggesting its association with the remodeling of the tumor ecological niche in HCC. Enrichment analysis showed that most CD8^+^ T cell subpopulations were significantly involved in chemokine-and natural killer-mediated cytotoxicity-related pathways, especially *HSPA1B* ^+^ Tcm, which was significantly enriched in HCC. This result suggested that the subpopulation may be immunologically active in the HCC microenvironment, aggregating to lesions and exerting immunocidal effects (Figure 6F).

GRN analysis of CD8^+^ T cells showed that the target genes were divided into three modules regulated by TFs including *THAP1*, *IKZF1*, *YBX1*, *FOXA2*, *NR2F1*, and *FOXP1* (Figures 6G, H). Pseudotime analysis showed that *HSPA1B* ^+^ Tcm was positioned at a late stage of development, suggesting that as HCC develops, this subpopulation of cells is stimulated by antigens, proliferates, and differentiates to exert antitumor effects (Figure 6I). Survival analysis revealed that among the subpopulation-specific markers, *BTG1* and *HSPA1B* had prognostic potential for HCC (Figures 6J, K). In conclusion, *HSPA1B* ^+^ Tcm subpopulation plays an important role in the immune response to HCC, suggesting that increasing the abundance of this subpopulation may help inhibit tumor growth and metastasis.

### 3.7 Cellular communication events in the HCC microenvironment

**FIGURE 7:**
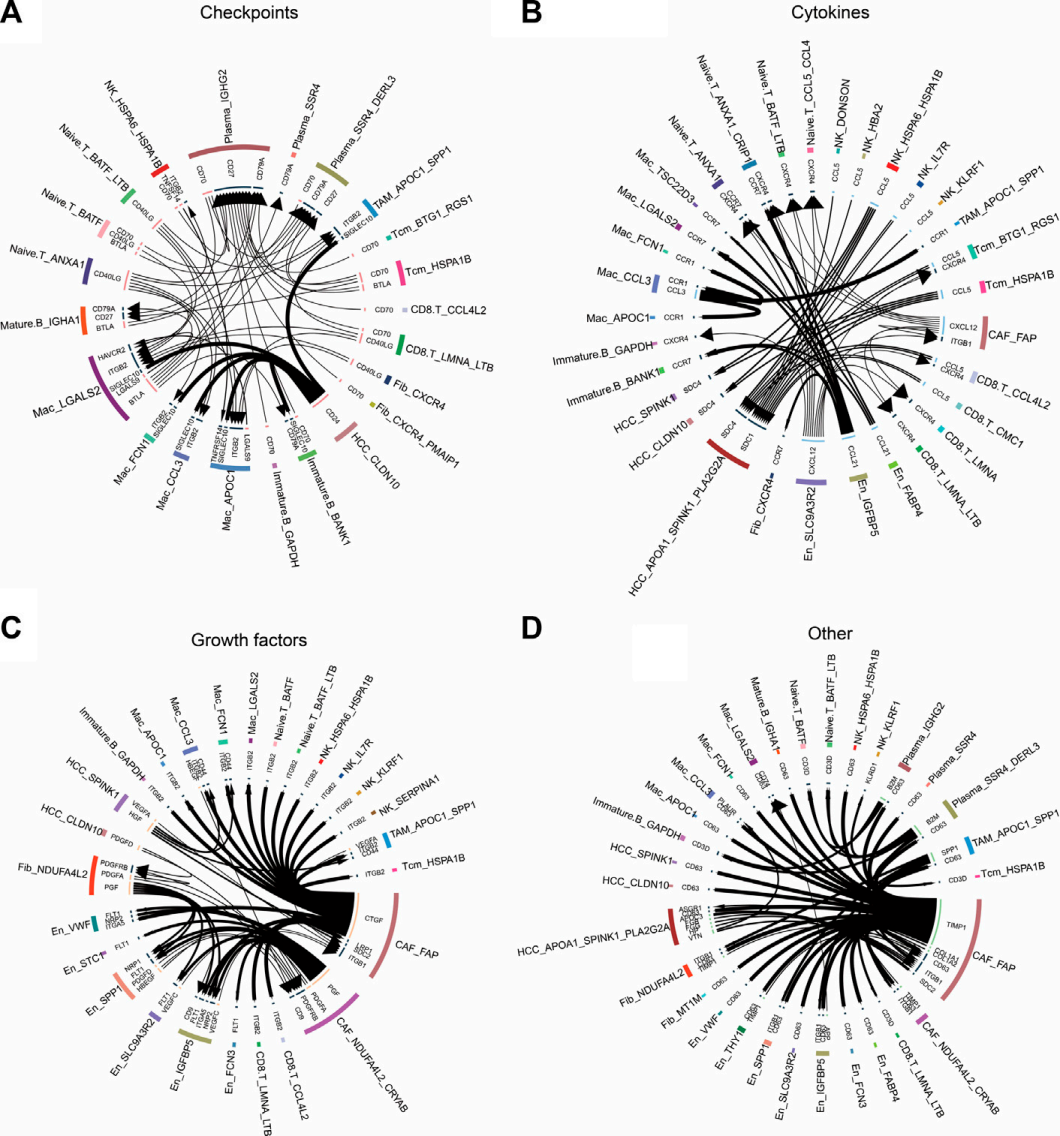
Circle plots demonstrate the ligand–receptor interactions between cells. **(A)** Receptor–ligand pairs related to immune checkpoints for each cell type. **(B)** Receptor–ligand pairs related to cytokines for each cell type. **(C)** Receptor–ligand pairs related to growth factors for each cell type. **(D)**. Other receptor–ligand pairs for each cell type, excluding immune checkpoints, cytokines, and growth factors.

To explore the relationship between non-tumor and tumor cells, we inferred their receptor–ligand interactions using the iTALK approach and constructed a cellular communication network divided into four modules: cytokines, growth factors, immune checkpoints, and other (Figure 7). We found that the CCL5 ligand secreted by *BTG1* ^+^ *RGS1* ^+^ Tcm and *HSPA1B* ^+^ Tcm cells bound to the SDC4/1 receptor on the surfaces of tumor cells (Figure 7B). Interestingly, the checkpoint CD24 ligand secreted by tumor cells bound to the SIGLEC10 receptor on macrophages to transmit inhibitory signals and reduce phagocytosis (Figure 7A). We also observed that tumor and endothelial cells released vascular endothelial growth factors, including VEGFC and VEGFA, as well as platelet-derived growth factors, including PDGFA and PDGFC (Figure 7C). Remarkably, in another module, the TIMP1 ligand secreted by CAFs bound to the receptor CD63 on the surfaces of tumor cells and TAMs, whereas the SPP1 ligand secreted by *APOC1* ^+^ *SPP1* ^+^ TAM cells bound to the ITGB1 receptor on CAFs (Figure 7D). These cellular communication networks reshape the tumor microenvironment and promote the development of HCC.

## 4 Discussion

In this study, we constructed a single-cell microenvironmental landscape of patients with primary HCC based on scRNA-seq data to reveal cell subpopulations with potentially specific functions in the HCC tumor microenvironment and to explore the interactions between HCC and the tumor microenvironment. High levels of CNVs are present in HCC tissue samples and have been suggested as potential factors in HCC development. Additionally, the significant increase in specific cell subpopulations compared to levels in controls may be related to HCC formation.

Previous studies have shown that elevated levels of *APOA1* are associated with liver injury (Gumilas et al., 2022). In the present study, *APOA1* was not only most highly expressed in the *APOA1* ^+^ HCC subpopulation, but was also highly expressed in other malignant HCC cells; therefore, it may be a potential biomarker for HCC. Pseudotime analysis suggested the *APOA1* ^+^ HCC subpopulation to be the origin of tumor development, which again demonstrates the potential of *APOA1* as a marker for early HCC detection. We found that HIF, a TF that induces hypoxic gene expression and helps tumor cells adapt to hypoxic environments, was highly expressed in the *APOA1* ^+^ HCC subpopulation (Balamurugan, 2016). In addition, we found that hypoxia-related genes, such as *ALDOB*, *GPC3*, *CP*, *PRDX5*, and *FBP1*, were highly expressed in the *DDX5* ^+^ HCC subpopulation. Hypoxia stimulates development of the EMT, and the CAF subpopulation in the present study also highly expressed *DDX5*. *DDX5* is a member of the DEAD-BOX family of RNA-unwinding enzymes, and its knockdown can promote migration, invasion, and EMT processes in HCC cells (Xue et al., 2018). Thus, these findings complement the potential mechanism underlying the oncogenic effects of *DDX5*.

Cell adhesion to the ECM is a key determinant among the myriad microenvironmental factors that influence drug resistance in cancer cells (Eke and Cordes, 2015). In the present study, we found that all fibroblast subpopulations were significantly involved in cell motility-related pathways, including the focal adhesion pathway. The abundance of the *NDUFA4L2* ^+^ Fib subpopulation was significantly higher in HCC. *NDUFA4L2* expression is positively correlated with tumor stage, and patients with higher expression have worse RFS than those with lower expression (Sarathi and Palaniappan, 2019). Pseudotime analysis showed that *NDUFA4L2* ^+^ Fib cells were positioned earlier on the differentiation trajectory than were *NDUFA4L2* ^+^ *CRYAB* ^+^ CAF cells, presumably because *NDUFA4L2* ^+^ Fib cells gradually differentiated into *NDUFA4L2* ^+^ *CRYAB* ^+^ CAF cells with the development of HCC. In addition, we found that *FAP* ^+^ CAF bound to CD63 in tumor cells by secreting the TIMP1 ligand. TIMP1 is a multifunctional protein that promotes cell proliferation, growth, survival, and differentiation and suppresses cell apoptosis in a variety of different tumor types (Justo and Jasiulionis, 2021); thus, the TIMP1–CD63 interaction between CAFs and HCC cells may promote the proliferation of HCC cells. Previous studies have shown that *FAP* ^+^ CAFs and *SPP1* ^+^ TAMs contribute to ECM remodeling and coordinate the formation of a prodesmoplastic microenvironment that prevents lymphocytes from infiltrating the tumor core (Qi et al., 2022). In our study, we found that SPP1 secreted by *APOC1* ^+^ *SPP1* ^+^ TAM cells bound to ITGF1 secreted by *FAP* ^+^ CAFs cells, suggesting that these two subpopulations may promote HCC development by remodeling the tumor microenvironment. Additionally, the higher abundance of naïve T cells in the HCC group than in the control group implied significant lymphoid immune infiltration of HCC tissues. *FAP* ^+^ CAF interacted with naïve T cells via the CXCL12–CXCR4 axis. CXCL12 is a homeostatic chemokine that regulates physiological and pathological processes such as inflammation, cell proliferation, and specific migration. TME enriched with CXCL12 is resistant to immune checkpoint inhibitor treatment (Portella et al., 2021). In this study, CAFs were the source of CXCL12 signaling, suggesting the importance of CAF research in the treatment of HCC with immune checkpoint inhibitors.

The *STC1* ^+^ En subpopulation was significantly enriched in the HCC group, with the highest abundance among the subpopulations identified in this study. Moreover, we found that high *STC1* expression is significantly associated with poor HCC prognosis. *VWF* promotes chronic hepatitis B virus- and hepatitis C virus-related HCC (Xiang et al., 2022). Another study showed that HCC is associated with significant changes in primary hemostasis, including increased platelet aggregation and elevated *VWF* levels (Zanetto et al., 2021). *VWF* participates in angiogenesis and negative regulation of angiogenic factors, which is an essential link in the growth, invasion, and metastasis of HCC. In the present study, the *VWF* ^+^ En subpopulation was significantly increased in the HCC group, suggesting that *VWF* may play a role in promoting angiogenesis in HCC.

The *APOC1* ^+^ Mac subpopulation was a major component of the macrophage ecology of HCC and was significantly more abundant compared to the levels in adjacent controls. *APOC1* expression was also higher in the *APOC1* ^+^ *SPP1* ^+^ TAM subpopulation, and this gene was specifically enriched in HCC lesions. It has been reported that *APOC1* is overexpressed in the TAMs of HCC tissues compared to those in normal tissues, and inhibition of *APOC1* promotes the transformation of M2 macrophages into M1 macrophages through the iron death pathway to reshape the tumor immune microenvironment (Hao et al., 2022), indicating the pro-cancer role of *APOC1* in HCC. Our study showed that *APOC1* was not only highly expressed in macrophages in HCC lesions, but also that macrophages with high *APOC1* expression were more abundant in HCC lesions than were other macrophages. Additionally, patients with high *SPP1* expression have poor prognosis, and *SPP1* is a pro-cancer gene in HCC (Liu et al., 2022). We found that *SPP1* was specifically expressed in TAM subpopulations, suggesting that it may function through macrophages. The *FCN1* ^+^ Mac subpopulation was significantly less abundant in tumor cells than in adjacent controls. *FCN1* is a member of the ficolin (FCN) family of proteins and a part of the innate immune system (Zhang et al., 2010). The results of the present study were consistent: lower *FCN1* expression was associated with a lower patient survival rate (Sun et al., 2022).

The *HSPA1B* ^+^ Tcm subpopulation was significantly increased in the HCC group, and *HSPA1B* was significantly enriched in all cell types in the HCC group, suggesting its involvement in remodeling of the tumor ecological niche in HCC. *HSPA1B* belongs to heat shock protein family A, where the binding of other heat shock proteins can stabilize existing proteins and mediate the folding of newly translated proteins in the cytosol and organelles (Faisal et al., 2022). Moreover, *HSPA1B* is involved in the ubiquitin-proteasome pathway through interaction with the AU-rich element RNA-binding protein 1, and the ubiquitin-proteasome system is an important non-lysosomal protein degradation pathway in cells that directly or indirectly affects the occurrence of various malignant tumors by regulating cell cycle activity and apoptosis-related proteins and activating or inhibiting the expression of proto-oncogenes and anti-oncogenes (Park et al., 2020). In addition, previous studies have shown that the receptor CCL5–SDC1/4 ligand interactions between regulatory T cells and tumor cells in pancreatic cancer promote tumor cell metastasis (Chen et al., 2022). The same intercellular communication signals were also observed in this study, where the CCL5 ligand secreted by *BTG1* ^+^ *RGS1* ^+^ Tcm bound to SDC4/1 receptors on the surfaces of tumor cells, suggesting that the interaction between *BTG1* ^+^ *RGS1* ^+^ Tcms and tumor cells via CCL5–SDC4/1 axis may promote tumor progression. These findings may provide new targets for HCC immunotherapy.

This study preliminarily revealed the cell subsets that lead to HCC in the tumor microenvironment based on scRNA-seq data; however, some limitations remain. First, only a single dataset was included in this study, and the sample size was relatively small. In addition, the conclusions of this study were mainly based on bioinformatics analysis; therefore, experimental validation with larger sample sizes is warranted.

In conclusion, our study suggests the presence of tumor cells with drug-resistant potential in the HCC microenvironment. Among non-tumor cells, high *NDUFA4L2* expression in fibroblasts may promote tumor progression, whereas high *HSPA1B* expression in Tcms may exert antitumor effects. In addition, there is significant immune infiltration into tumor lesions, and the interaction between *BTG1* ^+^ *RGS1* ^+^ Tcms and tumor cells via CCL5–SDC4/1 axis may promote tumor progression. Focusing on the role of CAFs and TAMs, which are closely related to tumor cells, in tumors would be beneficial to the progress of systemic therapy research.

## Data Availability

The original contributions presented in the study are included in the article/Supplementary Material, further inquiries can be directed to the corresponding authors.
