# Supplementary material for: Single-cell RNA sequencing reveals cell subpopulations in the tumor microenvironment contributing to hepatocellular carcinoma
Source: Front Cell Dev Biol. 2023 Jun 2;11:1194199. doi: 10.3389/fcell.2023.1194199 (PMC10272598; doi:10.3389/fcell.2023.1194199)
Supplement: Supplementary file 1 [file DataSheet1.docx]

Supplementary Material

Single-cell RNA sequencing reveals cell subpopulations in the tumor microenvironment contributing to hepatocellular carcinoma

**Running Title: Specific cell subpopulations of HCC**

**Jiamin Gao^1,^****^2,4,#^, Zhijian Li^1,2,6,#^, Qinchen Lu^1,2,3^, Jialing Zhong^2,3,^, Lixin Pan^1,2,3^, Chao Feng^1,2,3^, Shaomei Tang^2,3^, Xi Wang^2,3^, Yuting Tao^1,2,3^, Jianyan Lin^5*^, Qiuyan Wang^1,2,3,*^**

**^1^** Department of Biochemistry and Molecular Biology, School of Basic Medical Sciences, Guangxi Medical University, Nanning 530021, China.

**^2^** Center for Genomic and Personalized Medicine, Guangxi Medical University, Nanning, 530021, China.

**^3^** Guangxi Key Laboratory for Genomic and Personalized Medicine, Guangxi Collaborative Innovation Center for Genomic and Personalized Medicine, Nanning, 530021, China.

**^4^** Laboratory of Infectious Disease, The Fourth People’s Hospital of Nanning, Nanning 530023, China.

**^5^** Administrative Office, The First People’s Hospital of Nanning, Nanning 530016, China.

**^6^** Department of Clinical Laboratory, The First Affiliated Hospital of Guangxi Medical University, Nanning, 530021, China

**^#^**Contribute equally as co-first author.

**^⁎^ Correspondence:**

**Corresponding authors.**

Qiuyan Wang: Center for Genomic and Personalized Medicine, Guangxi Medical University, 22 Shuangyong Road, Nanning 530021, China. E-mail: [wangqiuyan@gxmu.edu.cn](mailto:wangqiuyan@gxmu.edu.cn).

Jianyan Lin: Administrative Office, The First People’s Hospital of Nanning, Nanning 530016, China. E-mail: linjianyan@126.com.

# Supplementary Figures

**
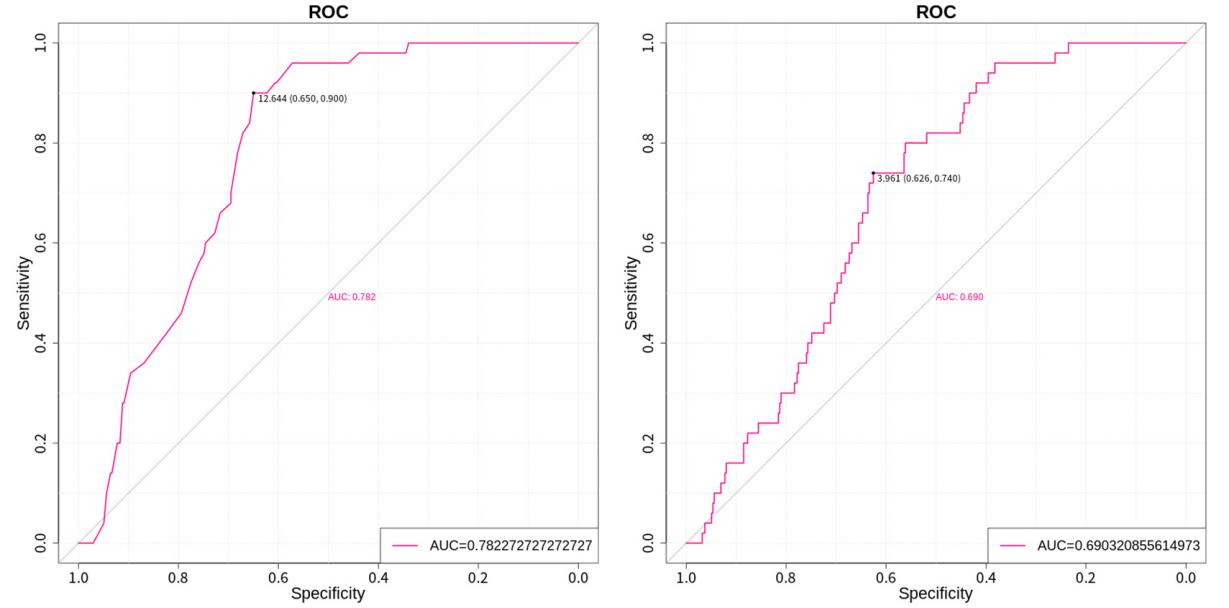
**

Figure S1. Receiver operating characteristic curves for *APOA1* and *SPINK1*.


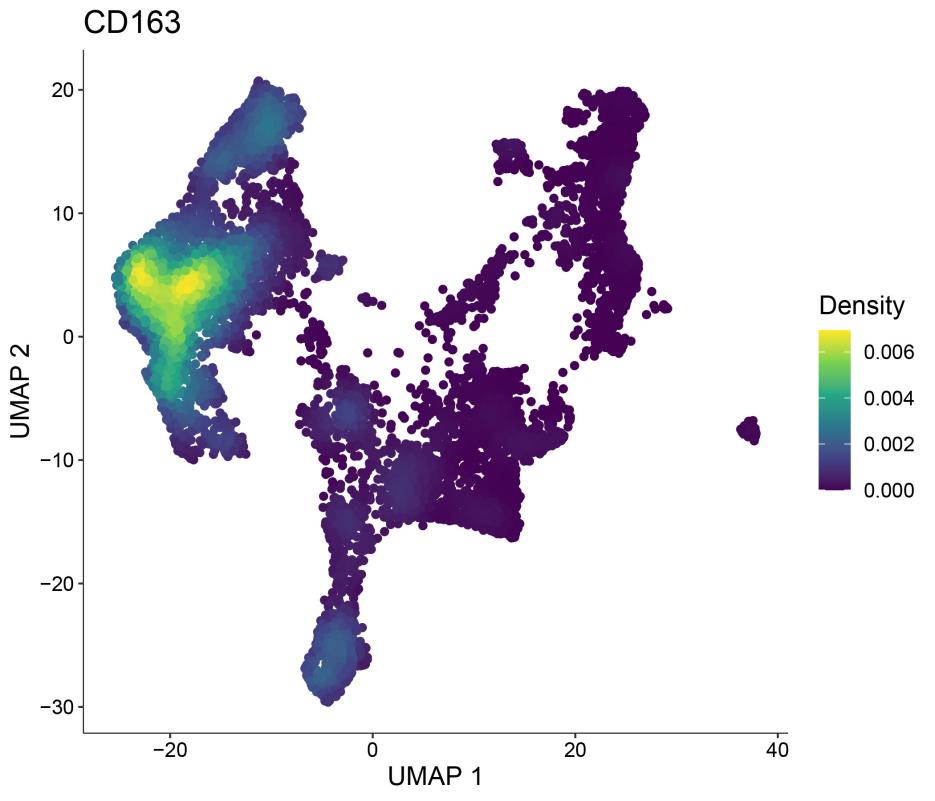


Figure S2. Density map showing *CD163* expression in macrophage subpopulations
